# *ClustalXeed*: a GUI-based grid computation version for high performance and terabyte size multiple sequence alignment

**DOI:** 10.1186/1471-2105-11-467

**Published:** 2010-09-17

**Authors:** Taeho Kim, Hyun Joo

**Affiliations:** 1 Laboratory of Systems Immunology, World Premier International Immunology Frontier Research Center, Osaka University, Suita, Osaka 565-0871, Japan; 2 Department of Physiology and Integrated Biosystems, College of Medicine, Inje University, Busan 614-735, South Korea

## Abstract

**Background:**

There is an increasing demand to assemble and align large-scale biological sequence data sets. The commonly used multiple sequence alignment programs are still limited in their ability to handle very large amounts of sequences because the system lacks a scalable high-performance computing (HPC) environment with a greatly extended data storage capacity.

**Results:**

We designed *ClustalXeed*, a software system for multiple sequence alignment with incremental improvements over previous versions of the ClustalX and ClustalW-MPI software. The primary advantage of *ClustalXeed *over other multiple sequence alignment software is its ability to align a large family of protein or nucleic acid sequences. To solve the conventional memory-dependency problem, *ClustalXeed *uses both physical random access memory (RAM) and a distributed file-allocation system for distance matrix construction and pair-align computation. The computation efficiency of disk-storage system was markedly improved by implementing an efficient load-balancing algorithm, called "idle node-seeking task algorithm" (INSTA). The new editing option and the graphical user interface (GUI) provide ready access to a parallel-computing environment for users who seek fast and easy alignment of large DNA and protein sequence sets.

**Conclusions:**

*ClustalXeed *can now compute a large volume of biological sequence data sets, which were not tractable in any other parallel or single MSA program. The main developments include: 1) the ability to tackle larger sequence alignment problems than possible with previous systems through markedly improved storage-handling capabilities. 2) Implementing an efficient task load-balancing algorithm, INSTA, which improves overall processing times for multiple sequence alignment with input sequences of non-uniform length. 3) Support for both single PC and distributed cluster systems.

## Background

Genetic and protein sequences are being discovered rapidly, and as a result, the number of sequences entered into biological databases is growing exponentially over time. Most of the work currently being done in computational biology involves searching for inter- and intra-sequence homology in massive volumes of genetic and protein sequence data, which are commonly based on a multiple sequence alignments (MSAs) [1]. However, increasing the computational efficiency to solve a variety of real MSA problems is still a challenging task because of the high demand for greater capacity and speed [2,3].

The oldest and most widely used MSA program that estimates trees as it aligns multiple sequences is ClustalW [4,5]. ClustalX is an integrated graphical-user-interface (GUI) version of the ClustalW multiple sequence alignment program [6]. It provides an easy-to-use work environment for performing MSA and pattern analyses. The latest version of ClustalX (version 2.0) added two new features [7]. The main advantage of ClustalX 2.0 is that it provides an easier way to maintain code for other applications. The new guided-tree implementation, compared with the older version, enables larger, faster computations. ClustalX 2.0 is now available for a number of platforms, including SUN Solaris, IRIX5.3 on Silicon Graphics, Digital UNIX on DECStations, Microsoft Windows for PCs, Linux ELF for x86 PCs, and Macintosh PowerMac.

Unfortunately, most of the currently available MSA programs are not suitable for large-capacity data storage and massive computation. These programs, including ClustalX (or X 2.0), are still single-PC based, and the storage and computation is entirely dependent on physical random-access memory (RAM). Past MSA performance evaluations focused simply on how compute-intense and sensitive the program was with respect to the longest-common-sequence (LCS)-based exact-string matching algorithm (e.g., the Smith-Waterman or Needleman-Wunsch method) [8]. Depending on both the volume of data to be aligned and the accuracy of the comparisons, computation using dynamic programming is extremely time-consuming when large sequence volumes and high accuracy are required simultaneously. Numerous parallel-computation programs, such as parallelized Praline [9], DiAlign P [10], ClustalW-MPI [11], and a commercial SGI parallel Clustal on a shared memory SGI multiprocessor [12], have been developed, primarily to increase computational speed, rather than for larger capacity data handling. Multiple sequence alignments with the Clustal series programs and the required features have been reviewed [13].

**Figure 1:**
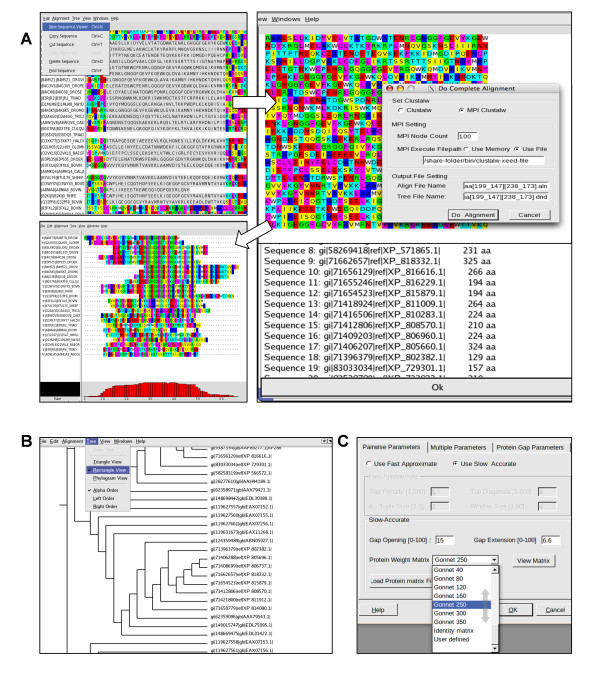
**Screen shots of *ClustalXeed***. The visual dialog boxes showing the powerful graphical user interface (GUI) environment for both parallel- and single-mode computations of multiple sequence alignments. (A) *ClustalXeed *enables easy iterative refinement of a poorly aligned region (shaded box area) on a new screen mode. (B) The enhanced tree drawing option provides three different scalable, high resolution vector graphics. This option is especially useful for generating phylogenies from large data sets. (C) Thirty-four different, ready-made protein weight matrices are helpful for detecting similarities among divergent or markedly biased sequences.

In this study, we report *ClustalXeed*, which was designed for a wide range of MSA purposes (Figure 1). This is a new ultra-high grid version of ClustalX supports both a large-scale MSA and efficient load-balancing. We also evaluated its overall performance using a typical grid-computation system. *ClustalXeed *is currently available for Linux 64-bit/32-bit platforms. A major point we emphasize is that *ClustalXeed *provides dual-mode sequence loading and computation platforms, where if the size of the biological sequence exceeds the available RAM, the user can readily shift to a distributed file-allocation (-disk memory) mode. The disk-storage system uses an idle node-seeking task algorithm (INSTA), which forces sleeping nodes into active modes during the massive file-swapping and pair-align computation to overcome slow computation speed. The GUI-based parallel-computation system is implemented with Linux and can be classified into two subsystems: the GUI engine (a highly modified ClustalX with an efficient load-balancing algorithm) for interactions between users and the parallel-computation system, and the pair-alignment engine for the calculation of pair-align matrices.

## Implementation

### Dual Computation mode: Single or Parallel Central Processing Units (CPUs)

*ClustalXeed *assigns two computation platforms incorporating physical RAM addressing, using a modified version of the *clustalw-xeed_mem *file, which is similar to the original ClustalW or ClustalW-MPI tools [5,11] and distributed file allocation, using the *clustalw-xeed *file. The new *clustalw-xeed *file enables large-scale disk memory storage and smart allocation of vast sequence data sets to a disk swap space for construction of temporary pair-align matrices and for accelerated computation. *ClustalXeed *uses a sequential file writing method that provides a straightforward and efficient way to read and write files. For a large number of sequences, *ClustalXeed *converts input sequences into individual sequence pairs and stores the pairs using the naming rule/tmp/xxxxx000P, where P = the pair sequence number and the generation number of the file name is always +1.

A distance matrix file, which is a single file with a file name of matrix-file080.tmp, is also generated in a disk storage unit and is based on all-to-all pair sets from the input sequence query. The calculated pair scores generated from each computation node are stored in the master node/tmp directory. Creation of this process involved modification of the three main programming functions in the original ClustalW (alloc_aln, pairalign_new.c, pairalign), as well as the pairalign message-passing-interface (MPI) algorithm in ClustalW-MPI [11].

For cluster analysis, *ClustalXeed *uses the distance matrix and the neighbor-joining clustering method to construct a similarity or guided tree. During this step, temporary changes in tree values are recorded sequentially by creating a new similarity tree file that contains the updated records at each computation stage. This technique provides an efficient file handling methodology for analyses involving frequent writing/reading of large data sets, as is required for dynamic programming using the sum of pairs (SP) scoring method. The similarity matrix files generated at each stage are named using the same naming rule as was described for the input file storage system. The final multiple sequence alignment (MSA) results are stored in a {*.aln} file for easy data retrieval.

### Dynamic Scan Load Balancing (DSLB)

**Figure 2:**
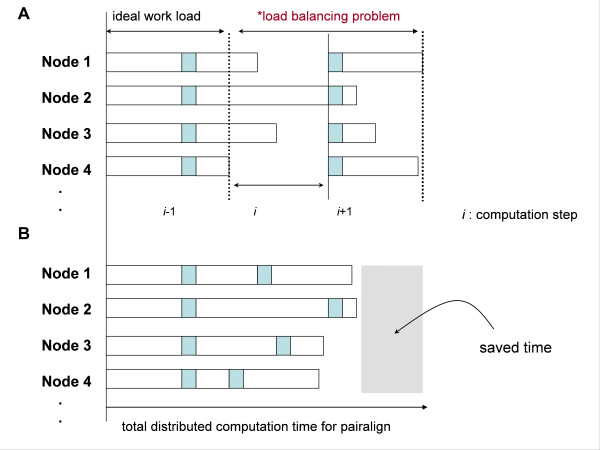
**General scheme of the load balancing problem**. (A) Workload imbalance condition. The overall computation performance will be reduced to the heavily loaded node if the number of sequence threads cannot be equally scheduled to the individual nodes. There will be one more thread on a heavily loaded node than on a lightly loaded node. (B) The idle node-seeking task algorithm (INSTA) overcomes load imbalance through timely assignment of new jobs to sleeping nodes, resulting in the equal distribution of work threads to all nodes.

Both ClustalW and ClustalX calculate pair-align scores to generate a guided tree for multiple sequence alignment. During this step, a distributed computation strategy may accelerate the computation. This means that the performance of any distributed parallel-computation approach depends on the balance of the workloads among the distributed nodes (Figure 2A). In an ideal case, the sequence of the elements to insert is uniformly divided between the work-node threads.

Depending both on the volume of the data to be aligned and the accuracy of the comparisons, computation by dynamic programming requires time-consuming iterations to achieve high accuracy. As an example, ClustalX has a parallelized version of ClustalX IRIX that is optimized to run on SGI Origin parallel computers running IRIX 6.5. The main problem of this parallelized version, however, is that the user has to spend extra time pre-sorting the input data to reduce load imbalances.

If the overall pair-align computation nodes are well balanced, performance will be greatly improved (Figure 2B). The effect becomes dramatic when input sequences dominate the computation time, due to excessively long or short strings. This is necessary in the case of highly parallel file-swap systems where load balancing is a key speed-up feature for performance enhancement. Physical RAM addressing is preferred for small-to-medium-sized sequences, whereas the file-swap (disk-storage) system is used for very large volume computations.

**Figure 3:**
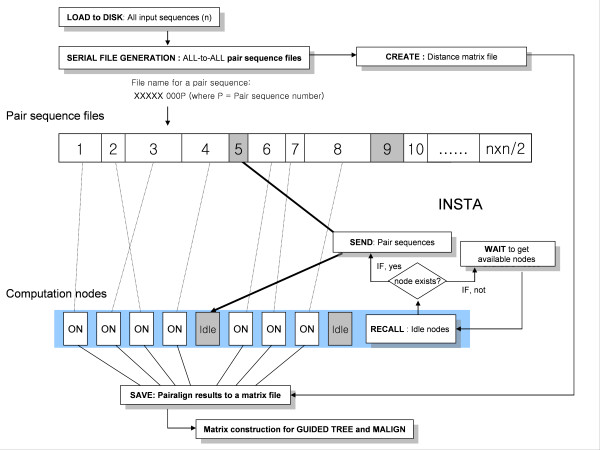
**Overview of the proposed multiple sequence alignment algorithm using a disk-storage system**. This algorithm differs from the conventional, fixed-size chunk scheduling used by ClustalW-MPI. With the new algorithm, the master allocates pair sequences individually to an appropriate compute node using the load balancer, INSTA. The embedded load balancer helps to identify an idle node for immediate task execution.

To overcome the slow speed of the file (-disk) memory computation, we designed an efficient load balancer, which uses a fast, intelligent scanning strategy to find sleeping computation nodes. Based on the previous distribution characteristics of ClustalW-MPI, we propose a dynamic scan-load balancing algorithm for efficient job-assignment of non-uniformly (unequally) distributed pair-align computation nodes, which is specified to increase the speed of interprocessor (nodes) communications (Figure 3). The original parallel version of ClustalW-MPI uses a fixed-size chunk scheduler algorithm to distribute sequence pairs to each node [14]. The main job of the master node in our system is to keep the slave nodes busy, as long as there is work to be done. That is, when a computation node completes its processing, it requests additional cue-sequence pairs from the master. This form of dynamic load-balancing continues until all of the sequences have been aligned. Once the job is submitted, it can be monitored and controlled via *ClustalXeed *main.

### New Features in *ClustalXeed *Core

Figure 1 shows the input panel for an MSA task. The mainframe of *ClustalXeed *is the same as that of ClustalX, because ClustalX is very familiar to PC-based MSA users. It provides a consistent interface, except for several new features for parallel computation, but the other functions are very similar to those in ClustalX.

#### New sequence editing options

*ClustalXeed *allows the user to change the order of sequences by simply cutting and pasting the sequence names. The sequence block grabber also provides a box-shaded area that enables the user to realign badly aligned sequences in a new window format, and to realign a small box region again. This option provides an independent task for the refinement of aligned sequences. The realignment range can be selected by simply clicking the mouse and dragging on the new sequence area (ClustalX and ClustalX 2.0 do not provide this). Easy Sequence finder enables a search for nucleic acids or proteins based on a partial sequence input.

#### Phylogenetic tree view Option Enhanced

For the convenience of direct tree-drawing, TreeView [15] open source was embedded because some environments cannot read "unweighted pair group method with arithmetic mean" (UPGMA) trees. This was a known problem in the previous versions of ClustalX and ClustalX 2.0. Users can save the resulting tree image as a postscript {*.ps} file. *ClustalXeed *supports standard TrueType and Postscript fonts, which may increase the resulting tree resolution when enlarged, especially in the case of a huge sequence data set.

#### Real-time in-process dialogue box

A real-time in-process dialogue box was built to enable the user to quickly monitor the current status of the computation, the progression of the job, and information on the number of nodes involved in the calculation. Neither ClustalX nor X2.0 gives feedback on the computation status. When the MSA is finished, the user can save all of the computation history to a {result.log} file. The used parameters are also saved in the working directory as a {result.par} file. This allows a user to view and edit tag information about all the individual batch sequence-alignment jobs.

#### Secondary structure prediction

The GOR IV [16] and PHD [17] options were added, although their installation requires permission from the original developer. We do not provide this permission in *ClustalXeed*.

#### Protein weight matrices

*ClustalXeed *provides more options for selecting protein-weight matrices. The former version of ClustalX (or 2.0) provides only three different protein-weight matrices: BLOSUM 30, PAM 350, and Gonnet 250. We added more than 34 different types of protein-weight matrices for specifying scoring tables for the easy and accurate adjustment of SP score improvement. This allows the user to reduce or increase the multiple sequence alignment sensitivity.

## Results and Discussion

*ClustalXeed *allows the alignment of large numbers of nucleic acid or protein sequences. Generally, the memory space requirement for the dynamic programming algorithm follows O (m × n). Based on this, for over 400000 sequences, MSA requires at least 1.28 terabytes of memory (i.e., 400000 × 400000 × 8/2) just to construct a distance-tree matrix, regardless of sequence length. We evaluated our software using large volumes of real nucleic acid and protein data sets to assess its overall and detailed performance. We set up a typical 100 × CPU AMD Opteron 244 (1.8 GHz) 64-bit cluster system with Linux (Fedora 4 Core). The master node consists of a 10-terabyte HDD (Samsung Electronics): 3ware RAID adapter (RAID 0, striping) and 8 GB DRAM, for a huge-tree matrix construction. Each node is a dual-core Opteron system with 4 GB DRAM and a 1-terabyte HDD.

To confirm the full sequence-loading and speed-up computation, almost six million sequences (23.08 GB) in an nt.gz file were first downloaded from ftp://ftp.ncbi.nih.gov/ blast/FASTA. These DNA sequences were divided into smaller data sets containing 150000 to 280000 sequences. With 200000 sequences as the first test set, which requires a nearly 8-terabyte file-managing environment for the MSA job, the elapsed computation time was 1.5- to 6-times shorter than that without INSTA. The total execution time for the complete alignment of 200,000 nucleic acid sequences by the disk-storage system was about 4700-6100 s with INSTA, and 7500-24000 s without INSTA. Once the INSTA was built, the overall speed-up rate was directly increased with the added computation nodes. The total computation efficiency depends on the pair-align scoring stage (i.e., the "bottleneck" step). For a given number of sequences, the average speed of *ClustalXeed *performance increased with the number of computation nodes (up to 50 nodes were tested).

**Figure 4:**
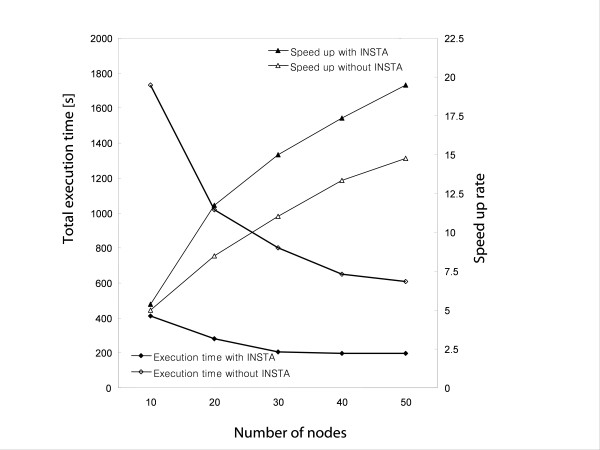
**The effect of using idle node-seeking task algorithm (INSTA) on sequence alignments of the cytochrome P450 superfamily, performed using the disk-storage algorithm**. For up to 40 nodes, the total execution time decreased as the number of computation nodes increased. In cases of conditions of load imbalance, most nodes were not busy. In an extreme case, 75% of the overall nodes were idle state during the computation. This observation indicates that INSTA increases turnaround frequencies of the idle nodes for assigning the next job; thus, INSTA ensures that most nodes are busy. Increased speed is the main performance criterion for parallel and distributed algorithms. Within this work, it is possible to predict the system size that is required to adjust cluster node numbers and thus maximize speed-up and optimize computing resource utilization.

As a second performance test, we extracted from NCBI a data set comprised of 52,750 protein sequences from the cytochrome P450 superfamily. The average length of the protein sequences was 440 amino acids (aa), with minimal length around 10 aa and maximal length of 11600 aa. In total, 1.39×10^9 ^(i.e., 52750 × 52749/2) pair-wise sequence alignments are required to complete each SP score calculation. We randomly mixed all of these sequences and aligned them using the disk-storage system. Figure 4 summarizes the obtained execution times of different job-balancing conditions as a function of node numbers. When the load-balancing algorithm was applied, the execution time of *ClustalXeed *was more than four times faster than the usual file-swap mode. The average speed-up for 20 nodes was 11.7 with INSTA and 7.5 without INSTA. The observed speed-up difference was due to the efficient load-balancing of sparsely distributed sequence pairs, and it clearly demonstrated the success of the implemented load-balancing algorithm. We showed that there exists an optimal node number to be assigned to jobs for the effective usage of computation resources. It is important to determine what system size will result in the maximum speed-up. The speed-up rate, as a function of participating node numbers, was not linear, because there was implicit waiting time due to communication latency (i.e., disk access time and network delay). All pairs of sequences in the *ClustalXeed *main disk-storage are transferred to compute nodes, and the calculated pair scores are then returned to the master and are saved in the disk-storage distance matrix (see Figure 3).

## Conclusions

ClustalW is the most widely used multiple sequence alignment program. The program uses a profile-based progressive alignment, with the alignment process divided into three steps: (1) distance matrix/pair-wise alignment, (2) guided tree generation, and (3) progressive alignment. However, ClustalW is not capable of estimating phylogenies and alignments for large data sets, because of slow computation times and limited memory usage. Alternatives to ClustalW include ClustalX (or X2.0), a graphical user interface (GUI)-based version of ClustalW, and ClustalW-MPI, a recently developed, parallel implementation version of ClustalW. In the latter tool, all three required computation steps are performed in parallel to reduce the total computation time. For parallel job scheduling, ClustalW-MPI uses a fixed-size chunk scheduler, which divides each job into uniform, fixed sizes. This method of batch job scheduling sometimes reduces the communication overhead required for a small and moderate number of sequences having very short lengths, such as ESTs (expressed sequence tags). However, it is not always effective at balancing the workload required for a large number of sequences having unequal lengths. Under these circumstances, the iteration for the next job scheduling is often delayed, which may result in high processor idle time [18]. Moreover, because ClustalW-MPI uses a RAM memory-storage mode, it has the inherent problem of a shortage of memory available for large-scale data sets.

*ClustalXeed *was designed to simultaneously address the dual needs of fast computation and large sequence data handling. Core changes with significant strides in supporting disk-memory access capabilities and enhanced job scheduling can tackle this bottleneck by enhancing both storage capacity and speed. As mentioned previously, ClustalW-MPI delivers fixed-size chunks to every compute node before the calculation. The batch scheduling mode needs high memory on each node, which may result in processor idling when the sequence lengths are not uniform. In contrast, the *ClustalXeed *disk storage algorithm does not send all pair sequences to the computation nodes and instead master allocates (distributes) the pair sequences individually to an appropriate node using a load-balancing idle node-seeking task algorithm (INSTA) to reduce the initial file transfer cost and slave node memory requirements. *ClustalXeed *also provides a fixed size chunk algorithm in the memory-storage mode for alignment of small-to-medium-sized sequences.

Because *ClustalXeed *was designed to work at the capacity of disk memory space, it has no inherent limitation on input sequence amounts. With a 64-bit compiler version, *ClustalXeed *works with sequence arrays containing up to 18 terabytes in disk-storage mode. In the disk memory operation, alignment speed was also improved by incorporating INSTA, which allocates or re-allocates tasks to idle nodes during pair alignment without the requirement for prior task information. In this way, INSTA determines from moment to moment which imbalanced nodes can be resolved immediately by allocating a new job. The main advantage of INSTA is its prompt scanning and decision-making without requiring decision complexity of rescheduling or reallocating tasks to resources at runtime, as occurs with static or other general dynamic load-balancing algorithms.

While setting up a new sequence batch job, the user has the option to choose an appropriate alternate computing environment using the graphical menu option. Other precise values of multiple sequence alignment tuning parameters can be set for each *ClustalXeed *menu link individually (similar to the old versions of ClustalX or X 2.0). Users can follow the progress of their alignment execution from the real-time in-process dialogue box. As indicated previously, the new parameter inputs, the easy-to-use realign option, the fully integrated graphical user interface, and the improved speed-up performance for very large volumes of sequence data may provide optimized computation of biological sequence alignments. *ClustalXeed *demonstrates that the embedded INSTA is a simple and effective means to meet the high computational demands (speed and memory) for massive biological-sequence alignments.

Moreover, large phylogenetic tree construction is becoming increasingly important, as vast amounts of biological sequences are used in the evolutionary and functional analysis of grouping organisms. *ClustalXeed *provides a great opportunity for the discovery of large and reliable phylogenetic trees between interesting biological sequence data sets. Based on the combination of 38 new protein-weight matrices, encouraging results show that *ClustalXeed *is one of most powerful MSA tools available for the rapid construction of huge phylogenies.

## Availability and Requirements

**Project name**: Clustal*Xeed*.

**Project home page**: http://pchm.inje.ac.kr/clustalxeed.

**Operating System(s)**: runs both on single and parallel Linux-based clustal systems.

**Programming Language**: C-language, Message Passing Interface (MPI).

**Other requirements**: described in the project home page. More detailed installation instructions and a tutorial manual will also be available for download.

**License**: *ClustalXeed *is available in source code, without fee, for academic, non-profit uses. The program is distributed in the hope that it will become further improved and put to practical use.

## Abbreviations

HPC: High Performance Computing; GUI: Graphical User Interface; MSA: Multiple Sequence Alignment; MPI: Message Passing Interface; RAM: Random Access Memory; INSTA: Idle Node-Seeking Task Algorithm; CPU: Central Processing Unit; HDD: Hard Disk Drive; I/O: Input/Output; MALIGN: Multiple Alignment; SP: Sum of Pairs

## Authors' contributions

HJ designed and wrote the software, with support from TK. Both authors contributed to the writing of the manuscript. All authors read and approved the final manuscript.
